# Preventive Conservation of Cultural Heritage: Biodeteriogens Control by Aerobiological Monitoring

**DOI:** 10.3390/s19173647

**Published:** 2019-08-22

**Authors:** Luigia Ruga, Fabio Orlandi, Marco Fornaciari

**Affiliations:** Department of Civil and Environmental Engineering, University of Perugia, Borgo XX Giugno 74, 06121 Perugia, Italy

**Keywords:** aerobiology, fungal spore, biodeterioration, preventive conservation, cultural heritage

## Abstract

Artefact conditions need to be continuously monitored to avoid degradation effects naturally caused by time and public exploitation in order to increase the value of cultural assets. In this way, the atmospheric analysis of both biological and chemical pollutants potentially present inside conservation environments represents valid support for the adoption of preventive conservation actions by evaluating periodically the presence of risk for the same artefacts. The aim of the present study was to analyze the fungal particles, potentially biodeteriogen, through aerobiological volumetric monitoring, particularly inside valuable historical, artistic, and cultural sites. Different exposition and conservation typologies of the artefacts with different flows of visitors were considered. The applied methodologies have furnished a reliable description of biological air pollution due to the presence of fungal spores—moreover, they have allowed for the prevention of risk situations and the measurement of their evolution in order to limit degradation processes. Through aerobiological monitoring, it was possible to provide important indications for interventions of prevention, conservation and restoration of cultural heritage in indoor environments.

## 1. Introduction

The effectiveness of the protection and conservation of cultural heritage derives from an appropriate level of safety, environmental control, management, care, and treatment of the exhibition environments for protecting the building from chemical and physical damage [1]. Cultural heritage preventive conservation plays a fundamental role as it allows the continuous control of the state of the artwork. The atmosphere normally contains, in addition to gaseous pollutants, various material and biological particulates—the latter is characterized not only by the presence of pollen grains, viruses, fragments of animal and plant origin, but also by fungal spores and the main bacteria responsible for the deterioration of the artefacts. It is necessary to promote and support the constant analysis and monitoring over time of all those environmental parameters that can give rise to various types of degradation, in particular that of a biological origin. The analysis of the biological component present in the air can contribute to defining an actual situation of risk of degradation and can provide important indications in case of preventive conservation actions [2]. Museum collections, the works conserved in ancient libraries, and in historical archives are as precious as they are vulnerable. Being mainly constituted by organic materials, these artefacts, in unfavourable environmental conditions, can represent a nutritional source and a physical support for micro-mushrooms and airborne bacteria, with the consequent irreversible deterioration (biodeterioration) of the artefacts. Fungal spores are the most frequent and harmful microorganisms associated with the biodeterioration of organic and inorganic materials in an indoor environment: their high metabolic versatility allow them to colonize various types of substrate (wood, paper, stone, etc.) with damage of both the aesthetic (initial step) and chemical/mechanical (destructive step) types [3,4].

Various vectors convey spores and “dangerous” concentrations are also variable depending on the species. Furthermore, some spores are highly allergenic for the museum “operators” and visitors. Conservation is therefore directed not only to the artwork, but also to their “container”, that is, to the environment that interacts with it. The “preventive conservation” must take into account the dynamic relationship that links the artwork to its context, and the biological characterization of the air is essential to make corrective interventions where situations of potential risk occur. In the management of cultural heritage it is therefore important to plan both indirect (prevention) and direct (conservation and restoration) interventions, for protecting the artefacts and allowing their usability. Priority must be given to prevention interventions for analysing the environmental conditions (temperature, relative humidity) of the sites where the works are exposed and air quality, both from a chemical and biological point of view (Aerobiological studies).

This study of organisms of biological origin dispersed and transported in the atmosphere and their successive effects on the exposed surfaces of different materials has become a relevant tool in the conservation of cultural heritage and in the evaluation of biodeterioration problems. In particular, aerobiological monitoring is essential for the establishment of a database necessary for the recovery phase of damaged materials (restoration), and in the prevention and conservation phases [5,6,7,8]. Aerobiological monitoring allows for the biological characterization of the air through two types of analysis: the qualitative type, for identifying the biodeteriogen species, and the quantitative type, that detects the atmospheric concentration of certain particles, thus dealing with the degree of contamination found in the environment. Furthermore, the choice of the most suitable sampler is fundamental to avoid over or under estimation of the environmental data [9].

Several studies were conducted in the city of Perugia (Umbria, Italy) in sites of particular historical, artistic and cultural value, with different exposition and conservation levels of the artefacts (museums, historical archives and ancient libraries) and also with different flow of visitors. The aim was to analyse, for the purposes of preventive conservation, the pollution level of the potentially biodeteriogen particles of fungal origin. Moreover, the risks of biological environmental contamination of the exposed and/or preserved organic material artefacts were considered to identify emission sources and to study the relationships between the factors affecting the degradation of cultural heritage. Another aim was to furnish the operators involved in the conservation and protection of cultural heritage with indications for the prevention, conservation and restoration acts to optimize protection procedures, and for the assessment of users’ health risks.

## 2. Materials and Methods

The studies were conducted monitoring the aerobiological particles within three sites: the conservative spaces of the Historical Archive of the Saint Peter’s Abbey in Perugia, the University of Perugia Doctorate Library and the museum environments of the National Gallery of Umbria during an important pictorial review, an event characterized by a considerable flow of visitors.

**Figure 1:**
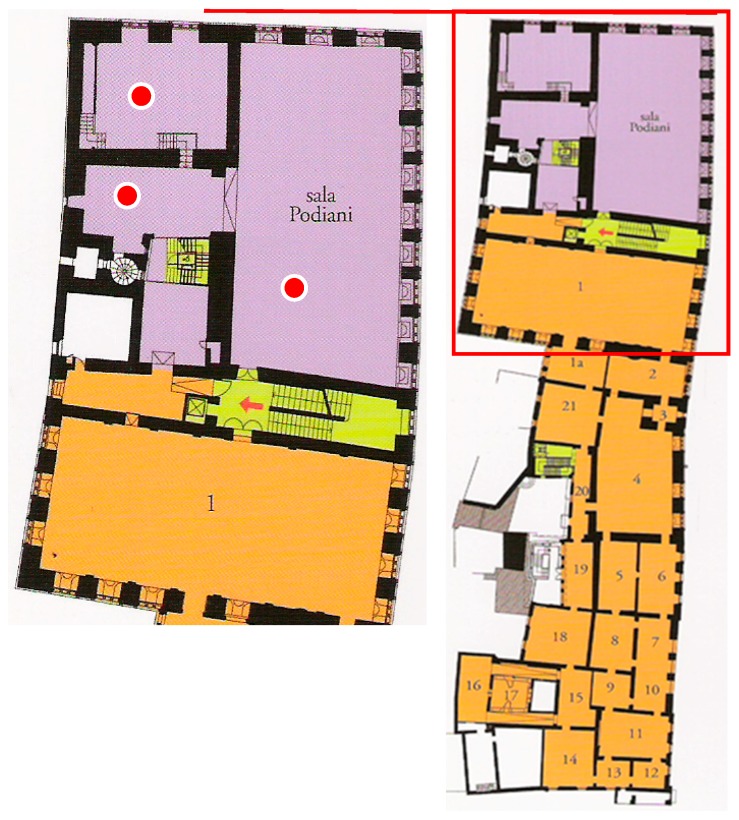
Third floor of the National Gallery of Umbria with focus on the sampling points (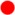) and entrance/exit ways.

Aerobiological monitoring was defined in relation to the most critical environments, to the areas where the particularly fragile artefacts are kept, where the anthropic impact is high (users and visitors disturb the environmental conditions, mostly if numerous and concentrated in small environments) and in relation to the exhibition and conservation rooms at greater biodeterioration risk (the rooms located near the entrance or exit ways). The sampling points inside one of the more representative rooms of National Gallery of Umbria are shown in Figure 1 evidencing the main entrance/exit.

### 2.1. Studied Areas

The Historical Archive of the Saint Peter’s Abbey in Perugia is located in a wing of the vast abbey complex and entrusted to the custody of the Benedictine monks. This heritage The Book of Patrimony, of great importance for the history of the city and of the Umbrian territory, is constituted by about 1730 books composed between the 11th–19th centuries. The archive is divided into three rooms, two of which are also used as consultation and study rooms. The third room is used as a warehouse for the preservation of the goods. The sampling was conducted for eleven months, within two weeks of each other, examining the two consultation rooms.

The Doctorate Library, named “Doctorate Hall”, represents one of the most precious places of the University for its frescoes and for the book heritage preserved in it, over 9000 pieces between the 16th and 19th centuries mainly theological and humanistic. The library is divided into two rooms: The Atrium or Entrance and the Major Room. The Atrium represents a sort of smaller-sized vestibule and precedes the Major Room [10]. Monitoring was conducted in the Atrium and in the Major Room (named Hall). Furthermore, as the “Hall” was distributed on two height levels, the aerobiological sampling was realized on both levels and the surveys were carried out for twelve months, within two weeks of each other.

The National Gallery of Umbria is one of the most prestigious and important collections in central Italy, with works of international interest between the 13th–19th centuries—since 1878 it has been housed on the upper floors of the “Palazzo dei Priori”, built starting from 1292 and enlarged several times [11]. In order to carry out the monitoring, some rooms were identified to represent the entire museum itinerary set up for the pictorial review, sampling was performed weekly during the pictorial review dedicated to the Italian painter “Pintoricchio”.

These expositive areas are equipped by an air-forced system managed by a dedicated software controlling the central heating and cooling systems.

The reduction of microbial and polluting substances suspended in the air coming from outside is achieved by passing through special antibacterial liquid filters. The system is controlled by detection probes connected with air conditioners in each room.

### 2.2. Aerobiological Sampling

Aerobiological sampling was conducted using non-invasive instruments to capture the fungal spores dispersed in the atmosphere by aspiration of known and constant volumes of air (volumetric method). In all the study sites the indoor environments were monitored and for comparison the external air nearby the considered buildings. A large part of the biological pollutants derived from the external environment, therefore, in the risk assessment, outdoor pollution should also be considered [12].

The objectives of this type of investigation were achieved mainly through the study of the airborne fungal components that were non-culturable and culturable (viable particles). A personal volumetric air sampler (Burkard Company Ltd., Rickmansworth, Hertfordshire, England) for glass slides based on the original Hirst model [13] operating for air depression impact was utilized for evaluating the non-culturable airborne fungal component. The spore trap aspire nominal air volumes (it was set to aspire 10 L/min for 10 min, reaching 0.1 m^3^ of sampled air), furnishing fungal spore concentration values (number of spores/m^3^) to rapidly estimate microbial air contamination.

Moreover, a three-stage (stage 1: >7.0 μ; stage 2: between 3.3 and 4.7 μ; stage 3: between 1.1 and 2.1 μ) Andersen impact sampler was utilized [6,8] to identify the potential presence of viable/culturable fungal spores in the atmosphere. This analysis was conducted through Petri dishes in the 3 stages contained Sabouraud Dextrose Agar with chloramphenicol to limit bacterial growth [14]. The aerobiological analysis was set to 10 min (at 28.3 L/min) for a total of 0.283 m^3^ of sampling air per week.

The viable component allows for the measurement of the spore concentration in the air that could deposit sediment on the artworks, and cause biodeterioration if the environmental conditions were optimal for their development. The analysis of the airborne culturable (viable) fungal component has been carried out only in the indoor spaces, while the monitoring was conducted utilizing contemporary the combined two samplers.

### 2.3. Identification of Fungal Groups

The fungal monitoring allowed us to capture spore and their successive development in fungal colonies was exploited to identify their genus by macro and micro recognition of morphological characteristics also through spore images databases [15]. The personal volumetric air sampler was stained with lactophenol blue solution and observed through optical microscope (magnification 400×). The Petri dishes were incubated at 28 ± 2 °C and after an incubation of three and six days, the developed microbial colonies were observed through a stereo-microscope. Then the slides were prepared with lactophenol blue solution and observed under optical microscope (magnification 400×) arriving to express CFU (colony-forming unit) per volume (CFU/m^3^).

### 2.4. Measurement of Temperature and Relative Humidity

Temperature and relative humidity values were recorded during biological sampling to have an indoor and outdoor environmental evaluation utilizing the digital thermo-hygrometer (RTGR328N Thermo/Hygro Sensor, Oregon Scientific, Tualatin, OR, USA), cable free, RF frequency 433 MHz.

### 2.5. Statistical Analysis

Monthly and weekly averages of the spore concentrations in the monitored sites and outdoor were utilized for charts realization. Moreover, to identify potential relationships between the concentrations of the indoor and outdoor non-culturable particulates with visitor flow, variable monthly averages were analyzed using statistic descriptive techniques (Pearson’s Correlation Coefficient and others).

## 3. Results

### 3.1. Historical Archive of the St. Peter’s Abbey

In the conservation environments of the monitored sites, and in the external atmosphere, the presence of particulate matter of non-cultivable and cultivable fungal origin was detected, whose concentration (spores/m^3^ and CFU/m^3^) was different depending on the construction characteristics of the monitored sites (Museum, Library, Archive) and according to the seasonal trend. Furthermore, in all the sites, through the qualitative analysis of the sampled particles, the presence of biodeteriogen spores was identified.

**Figure 2:**
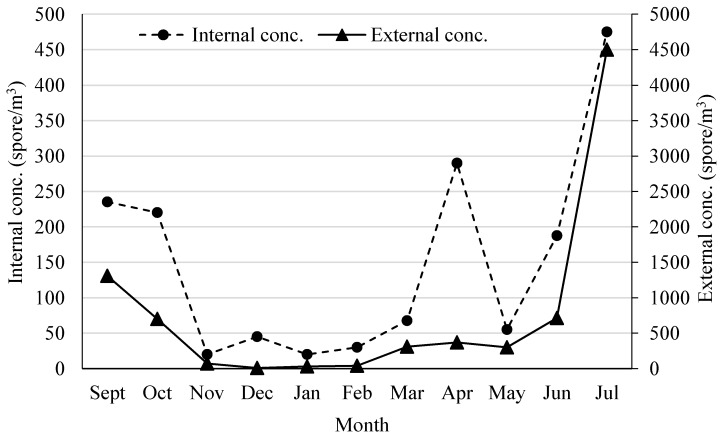
“Historical Archive of the St. Peter’s Abbey in Perugia”, the average monthly concentration of the biodeteriogen spore, sampled with the personal volumetric air sampler (non-cultivable fraction) both inside and outside.

Specifically, as regards the “Historical Archive of the St. Peter’s Abbey in Perugia”, the average monthly concentration of the biodeteriogen spore, sampled with the personal volumetric air sampler (non-cultivable fraction) both inside and outside (Figure 2), showed quite similar variations throughout the examined period. Both concentrations in fact show an overall increasing trend from the end of winter to full summer, reaching the highest values in July with 475 spores/m^3^ inside the archive and with 4500 spores/m^3^ outside. The results show a high correlation (r = 0.86) between the concentrations recorded around the monitoring site and in the internal archive environments. The qualitative analysis of non-cultivable particulate allowed to detect the presence of different fungal species both indoors and outdoors. In the archive areas the following species have been identified: *Alternaria* spp., *Bipolaris* spp., *Chaetomium* spp., *Cladosporium* spp., *Fusarium* spp., *Microsporum* spp., *Stemphylium* spp. and *Xilaria* spp. The monthly presence of fungal genus (non-cultivable fraction) is reported in Table 1. The most represented genus was the *Cladosporium* (from 44.4% to 100%) in all the surveys.

The sampling carried out with the Andersen sampler allowed us to improve the investigation. The results obtained from the quantitative analysis of the particulate matter detected with the mentioned sampler showed that the fungal average per cubic meter of air is also very variable and closely related to the detection period increasing in the spring and summer period. The qualitative analysis permitted to identify the presence and viability of spore groups, potentially biodeteriogen, in addition to those already identified on the slides obtained by sampling with the personal volumetric air sampler (Table 1). The following species were found: *Alternaria* spp., *Aspergillus* spp., *Botrytis* spp., *Chaetomium* spp., *Cladosporium* spp., *Coniothyrium* spp., *Humicola* spp., *Penicillium* spp., *Stachybotrys* spp., *Trichoderma* spp., *Trichophyton* spp., *Verticillium* spp. The most represented genera were *Aspergillus*, *Cladosporium* and *Penicillium*.

### 3.2. Doctorate Library (“Doctorate Hall”)

**Figure 3:**
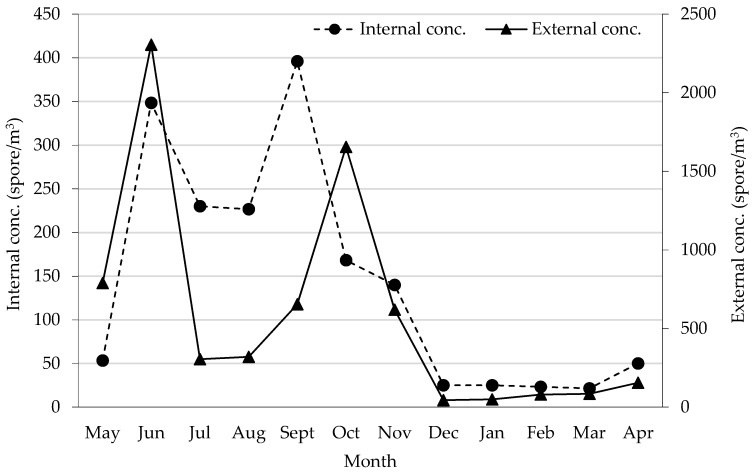
Mean spore concentrations both inside the Doctoral Library and in the external environment.

**Figure 4:**
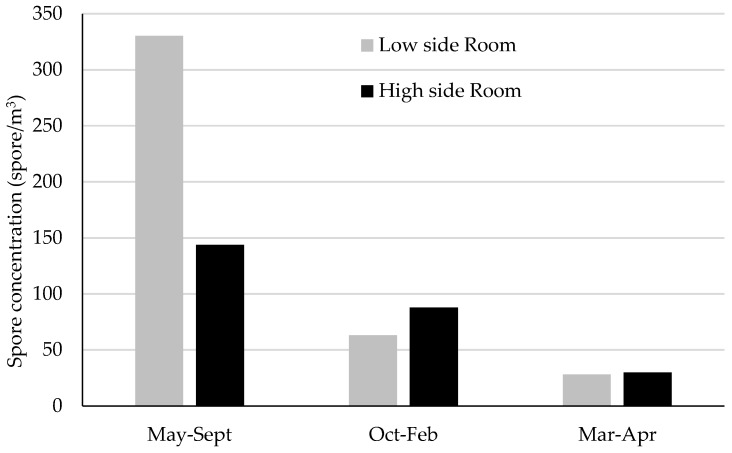
Mean spore concentrations in different periods (from May to September; October–February; March–April) monitored in low and high sides of the Doctoral Library Room.

As for the “Doctorate Library”, from the analysis of the mean concentration data we can see similar trends of the non-cultivable particulate, both in the internal that in the external environment (Figure 3). Both concentrations are higher in the spring-summer season (and partially in autumn) decreasing in the winter season. The results show a high correlation (r = 0.72) between the concentrations recorded outside the monitoring site and the internal environments of the Library with the first probably affecting the values of internal concentrations. As regards the analysis of the concentration on the two height levels inside the Room, as can be seen in the Figure 4 the average monthly spore values were highly variable and with a different spore distribution according to the seasons. In particular, in the spring-summer period the major concentration is in the lower part of the room while it decreases in the upper part. In the autumn and winter periods, the aerosporological values are greater on the upper part of the hall, confirming the inversion of the vertical concentration gradient if the indoor environment is artificially heated. The qualitative analysis of the non-cultivable particulate allowed for detection of the presence of spores belonging to the genus *Alternaria, Bipolaris*, *Chaetomium*, *Cladosporium*, *Epicoccum*, *Fusarium* and *Stemphylium*. *Cladosporium* was the most frequently fungal genus monitored with a monthly presence between 66.7% and 100% (Table 2)

Average monthly spore concentrations (CFU/m^3^) showed a growing trend of viable particles present in the atmosphere in the spring and summer period. *Cladosporium*, *Penicillium*, *Aspergillus*, *Alternaria*, *Geotrichum*, *Scopulariopsis* and *Trichophyton* are the most represented genera, besides the presence, even if in a more reduced value, of species belonging to the genus *Acremonium*, *Aureobasidium*, *Microsporum*, *Paecilomyces* and *Trichoderma*.

### 3.3. National Gallery of Umbria

**Figure 5:**
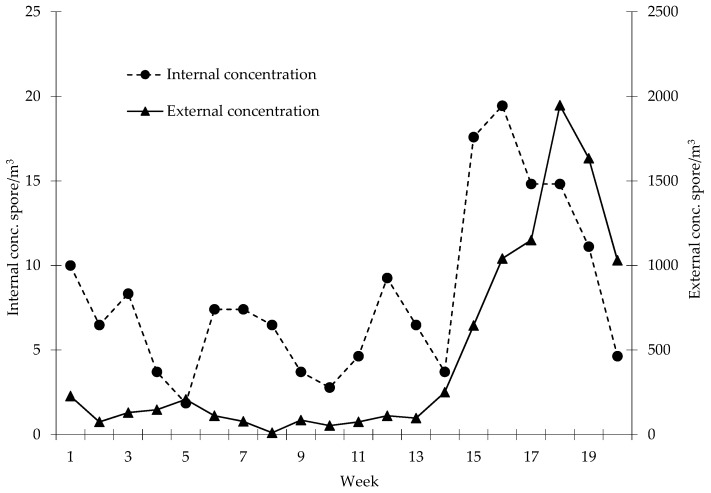
Week average of spore concentrations (spore/m^3^) inside National Gallery of Umbria and the external environment.

**Figure 6:**
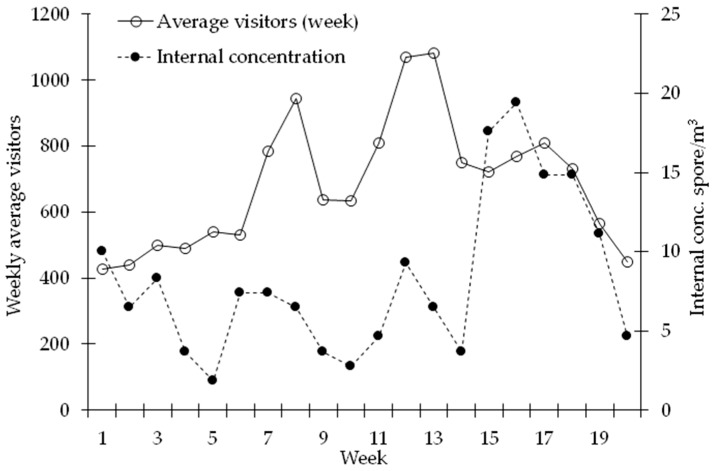
Week average of spore concentrations (spore/m^3^) and visitors (mean n°/week) inside National Gallery of Umbria.

Indoor and outdoor weekly average spore concentrations (potentially biodeteriogen spore per cubic metre) represented in Figure 5, in the “National Gallery” site, evidenced an overall positive trend. Both indoor that outdoor concentrations manifested an increasing trend moving to the spring-summer season. In the internal environment of the Gallery the maximum spore concentration value was recorded at the 16th week (19–25 May) with a value equal to 16 spores/m^3^. In outdoor environment, instead, the highest concentration was recorded at the 18th week (2–8 June) with 1947 spores/m^3^. Furthermore, the analysis of data concerning the average weekly concentrations of fungal spores (spores/m^3^) allowed to highlight a substantial difference between internal and external environment. In particular, the indoor concentrations show, on average during the studied period, a reduction of approximately 94% compared to outdoor. In Figure 6 the average trends of weekly internal concentrations and those related to the number of visitors are shown. The trend analysis evidenced the increase in attendance recorded up to the 18th week followed by a marked decline. As for the average values of internal concentration, the growing trend continues until the final monitoring phases, showing only a slight decrease in recent weeks.

The potential relationships between indoor, outdoor concentrations, and the flow of visitors firstly revealed a significant correlation between the two environmental concentrations expressed by a correlation coefficient equal to r = 0.63 during the entire study period (Table 3A). Bio-aerosol sampled inside the gallery exhibition rooms could largely derive from the exchange of air masses with the outside. As far as potential visitor influence is concerned, an elaborated variable was considered checking the interaction between visitor trends and external spore concentrations (VEC), created by multiplying the cited transformed variables (Visitors^2 * External conc.^0.5) for obtaining the best correlation with indoor spore concentrations. This elaborated variable showed highest correlations from the first to the twentieth week (r = 0.77) in comparison to single external concentration, reaching the highest values considering the restricted period between 9th and 20th week (r = 0.85) (Table 3B).

The qualitative analysis of the airborne fungal component sampled with the personal volumetric air sampler (non-cultivable fraction), allowed to identify weekly the presence of potentially biodeteriogen spores both in the internal environments of the gallery, and in the external environment.

The weekly percentage presence of fungal genus (non-cultivable fraction) is shown in Table 4. The most represented spore genus and most frequently monitored in the Gallery were the *Cladosporium*, the *Epicoccum*, the *Cephalosporium,* and the *Alternaria*. In particular, *Cladosporium* spp. was present in all the surveys, regardless of the monitoring season, with a presence range between 20–100%.

Furthermore, the examination of the data showed that the monthly presence in the outdoor air of the different biodeteriogen groups did not always coincide with their spread in the internal environments of the Gallery.

The sampling conducted with the Andersen sampler allowed to improve investigation also of this site. In correspondence of each survey, a constant presence of viable particles is observed and a weekly average increase of CFU/m^3^ for the whole study period. The largest number of colony forming units (CFU) was observed at the 17th week (26 May–1 June), with a total fungal value of 113 CFU/m^3^, a value attributable to a high presence of the genus *Cladosporium* and *Geotrichum* spores found mainly in May. The analysis of the viable airborne fungal component has allowed, also in this case, to more deeply ascertain the presence of various potentially biodeteriogen fungal groups, such as *Penicillium*, *Aspergillus*, *Alternaria*, *Trichoderma*, *Xylaria* and *Memnoniella*, in addition to those already identified with the personal volumetric air sampler. *Cladosporium*, *Penicillium* and *Aspergillus* resulted with the Andersen method, the most commonly spore represented and the most frequently monitored genera.

## 4. Discussion

The simultaneous use of both volumetric samplers, the Burkard personal volumetric air sampler and the Andersen, is important because they allow for the detection of the spores concentration in the atmosphere, measuring the bio-contamination with different technical characteristics but providing an evaluation of the potential biodeterioration of cultural heritage in indoor environments. The personal volumetric air sampler allows to quickly detect fungal spores, or the potentially viable and the non-viable fraction. This is important because non-viable particles can also represent a risk for artworks and for people’s health [16]. The Andersen sampler permits to ascertain the presence of various potentially biodeteriogen fungal groups, in addition to those already identified by the first Spore Trap and to verify above all the presence of viable spores through the development of different fungal colonies on the culture substrate. The use of different volumetric samplers allows for the sampling of a wider spectrum of fungal airborne spores, standardizing the sampling methodology in the conservation environments where the results are often difficult to compare due to the different techniques adopted [16]. The quantitative evaluation of airborne sampled particulates supports growing trends, both for the non-cultivable fraction and for the viable fraction, from the winter season to the spring–summer and autumn season, which is probably linked to the seasonal trends.

The maximum concentrations of fungal spore in Perugia area characterized by a transitional climate character from temperate to Mediterranean can be recorded in summer and autumn in correspondence with the highest humidity values [17,18]. June and July were the months with the highest concentration values considering that fungal growth increase with high humidity levels and air temperature between 18–32 °C, environmental conditions frequently recorded during summer periods in central Italy [19,20,21,22,23].

The quantitative values of indoor spore concentrations were different, being influenced by the seasonal period as well as the different environmental typology (the National Gallery is equipped with forced ventilation whereas the Doctoral Library and the Historical Archive are characterized by natural ventilation) that can affect the biological particulate matter amounts. In particular, the concentrations recorded in the internal environment of the Gallery show on average, for the entire period studied, a reduction of about 94% of biodeteriogen spores, compared to outdoor data. However, even if the outdoor concentrations influence those indoors, the presence of the air conditioning system in the gallery worked perfectly, causing a notable decrease in the diffusion of fungal spores in the air of its exhibition spaces.

The different microclimatic conditions, and in particular the ventilation (due to air conditioning systems or to the windows opening), can justify the different level of contamination in indoor environments [24]. Moreover, the regulation of the temperature and relative humidity variables in cultural heritage conservation environments is very important as such factors can favour or accelerate the processes of deterioration of the artworks’ constituent materials [3,25,26].

The large number of visitors linked to the increasing trend of outdoor spore concentrations during summer determined indirectly a pollutant increase that is potentially dangerous for the artworks. Nevertheless, the interaction between visitor numbers and outdoor pollutants does not seem to improve particularly internal concentrations, probably thanks to the presence in the gallery of an efficient air conditioning system. In this sense, in all the different monitored sites, external spore concentrations presented higher variability in comparison to those recorded inside exposition rooms. Average coefficients of variation of spore concentrations captured outdoors were double those of the indoor ones, proving the high seasonality trend of spore presence in the outdoor environment and the effects of air conditioning system to control spore level inside the monitored rooms.

Indoor and outdoor aerobiological monitoring by a qualitative point of view realized through volumetric sampling methodologies allowed us to identify several fungal genera eventually dangerous for artifacts representing potentially biodeteriogen particulates. Archive materials, documents, and works of art made from organic materials preserved in museums may be more or less damaged by biodeteriogen microfungi [12,27,28,29,30,31].

Furthermore, in all the studied sites, qualitative analysis has allowed to detect the presence of one or more fungal species in the indoor samples and their absence in the outdoor ones, suggesting the presence of other contamination sources in the internal conservative sites.

## 5. Conclusions

The combined aerobiological sampling methods have provided a reliable description of biological air pollution. The identification of potentially biodeteriogen particles and their degree of variability over time was allowed to periodically check the state of conservation of the artefacts. Moreover, volumetric sampling methods permitted to evaluate the areas with the greatest biological pollution and with the most sensitive conservation environments to identify principal potential environmental risks of contamination. A periodic aerobiological monitoring program can provide, to the subjects involved in the protection of cultural assets, useful indications and contributions to develop preventive intervention strategies for the safeguarding, rational management, and conservation of artworks in indoor environments. In restoration works, biological monitoring can provide more precise indications on the possible causes of alteration of the materials that constitute the physical support of the artworks, also providing important information for the assessment of health risks for users. It would be important to have the opportunity to conduct these studies in many more sites in order to deepen our understanding of the interaction between environmental factors and indoor airborne spores, or to identify new ones, but above all to increase the availability of aerobiological data and knowledge. This could improve the definition of guidelines in order to standardize environmental monitoring protocols, the latter necessary to promote and support preventive conservation, as well as representing a base of knowledge and information indispensable to the subjects involved in the design and establishment of new conservation sites and museum spaces.

## Figures and Tables

**Table 1:** Monthly percentage presence of fungal genus (non-cultivable fraction), inside the Historical Archive of the St. Peter’s Abbey in Perugia.

	Month										
Genus	Sept	Oct	Nov	Dec	Jan	Feb	Mar	Apr	May	Jun	Jul
Alternaria	−	−	−	−	−	−	6.7	−	−	5.3	4.2
Bipolaris	−	−	−	−	−	−	−	−	−	5.2	−
Chaetomium	10.4	12.8	−	−	−	−	13.3	−	−	−	1.0
Cladosporium	87.5	87.2	80.0	44.4	85.7	83.3	80.0	96.1	100.0	84.2	93.8
Fusarium	−	−	−	−	−	−	−	3.9	−	−	1.0
Microsporum	2.1	−	−	−	−	−	−	−	−	−	−
Stemphylium	−	−	−	−	−	−	−	−	−	5.3	−
Xylaria	−	−	20.0	55.6	14.3	16.7	−	−	−	−	−

**Table 2:** Monthly percentage presence of fungal genus (non-cultivable fraction), inside Doctorate Library.

	Month											
Genus	May	Jun	Jul	Aug	Sept	Oct	Nov	Dec	Jan	Feb	Mar	Apr
Alternaria	16.7		4.2	5.6	−	−	−	−	−	−	−	−
Bipolaris	−	2.7	−	−	−	−	−	−	−	−	−	−
Chaetomium	−	3.0	4.2	−	−	−	−	−	33.3	−	−	−
Cladosporium	83.3	90.6	91.7	93.4	99.0	94.1	91.3	100.0	66.7	100.0	100.0	100
Epicoccum	−	1.0	−	1.0	1.0	−	1.0	−	−	−	−	−
Fusarium	−	2.7	−	−	−	−		−	−	−	−	−
Stemphylium	−	−	−	−	−	5.9	7.7	−	−	−	−	−

**Table 3:** Correlation of weekly averages of visitors, internal and external concentration spore (IC, EC) and interaction effects between visitors and external spore (VEC).

**From 1st Week to 20th (A)**				
	**IC**	**EC**	**Visitors**	**VEC**
IC	1.00			
EC	0.63	1.00		
Visitors	0.20	−0.07	1.00	
VEC	0.77	0.79	0.43	1.00
**From 9th Week to 20th (B)**				
	**IC**	**EC**	**Visitors**	**VEC**
IC	1.00			
EC	0.62	1.00		
Visitors	0.12	−0.40	1.00	
VEC	0.85	0.73	0.25	1.00

IC = Internal conc.; EC = External conc.; Visitors = weekly average; VEC = ”Visitors” X “EC”.

**Table 4:** Weekly percentage presence of fungal genus (non-cultivable fraction), inside National Gallery of Umbria.

	Week																			
Genus	1	2	3	4	5	6	7	8	9	10	11	12	13	14	15	16	17	18	19	20
Alternaria	−	−	−	−	−	−	−	−	−	33.3	−	−	28.6	−	−	4.8	11.1	−	−	−
Botrytis	−	−	−	−	−	−	11.1	−	−	−	−	−	−	−	−	−	−	−	−	−
Cephalosporium	−	−	30.0	16.7	−	−	−	−	−	−	−	16.7	−	−	−	38.1	−	−	−	−
Chaetomium	−	−	−	−	−	−	−	−	25.0	−	−	33.3	−	−	−	−	−	6.3	−	−
Cladosporium	100.0	100.0	70.0	50.0	20.0	100.0	55.6	62.5	75.0	66.7	100.0	50.0	57.1	75.0	100.0	57.1	72.2	93.8	83.3	100.0
Epicoccum	−	−	−	−	40.0	−	11.1	12.5	−	−	−	−	14.3	25.0	−	−	16.7	−	−	−
Ophiostoma	−	−	−	−	40.0	−	−	−	−	−	−	−	−	−	−	−	−	−	−	−
Stachybotrys	−	−	−	33.3	−	−	−	−	−	−	−	−	−	−	−	−	−	−	−	−
Stemphylium	−	−	−	−	−	−	−	25.0	−	−	−	−	−	−	−	−	−	−	16.7	−
Trichoderma	−	−	−	−	−	−	22.2	−	−	−	−	−	−	−	−	−	−	−	−	−

## References

[1] Koestler R.J., Koestler V.H., Charola A.E., Nieto-Fernandez F.E. (2004). Art, Biology, and Conservation: Biodeterioration of Works of Art.

[2] Ruga L., Bonofiglio T., Orlandi F., Romano B., Fornaciari M. (2008). Analysis of the potential fungal biodeteriogen effects in the “Doctorate Library” of the University of Perugia, Italy. Grana.

[3] Sileo M., Gizzi F.T., Masini N., Gizzi F.T., Masini N. (2013). Monitoraggio microclimatico: Passato, presente e prospettive future. Salvaguardia, Conservazione e Sicurezza del Patrimonio Culturale. Nuove Metodologie e Tecnologie Operative.

[4] Ruga L., Orlandi F., Romano B., Fornaciari M. (2015). The assessment of fungal bioaerosols in the crypt of St. Peter in Perugia (Italy). Int. Biodeterior. Biodegrad..

[5] Mandrioli P., Zanotti Censoni A.L. (1982). L’aerobiologia degli spazi confinati di interesse artistico. Boll. D’arte Ser. Spec..

[6] Sorlini C. (1993). Aerobiology: General and applied aspects in the conservation of art works. Aerobiologia.

[7] Nugari M.P., Roccardi A. (2001). Aerobiological investigations applied to the conservation of cultural heritage. Aerobiologia.

[8] Nugari M.P. (2003). The aerobiology applied to the conservation of works of art. Biohazard Restor..

[9] Mandrioli P., Caneva G., Sabbioni C. (2003). Cultural Heritage and Aerobiology: Methods and Measurement Techniques for Biodeterioration Monitoring.

[10] Vermiglioli G.B. (1843). Cenni Storici Sulle Antiche Biblioteche di Perugia.

[11] Garibaldi V., Bon Valsassina C., Garibaldi V. (1994). La Galleria Nazionale dell’Umbria. Dipinti, Sculture e Ceramiche della Galleria Nazionale dell’Umbria, Studi e Restauri.

[12] Caneva G., Nugari M.P., Salvadori O. (2005). La Biologia Vegetale per i Beni Culturali, Vol. I, Biodeterioramento e Conservazione.

[13] Hirst J.M. (1952). An automatic volumetric spore trap. Ann. Appl. Biol..

[14] Booth C. (1971). Methods in Microbiology.

[15] Saenz Lain C., Monserrat Gutierrez Bustillo A. (2006). Esporas atmosfericas en la Comunidad de Madrid.

[16] Pasquarella C., Sansebastiano G.E., Saccani E., Ugolotti M., Mariotti F., Boccuni C., Signorelli C., Fornari Schianchi L., Alessandrini C., Albertini R. (2011). Proposal for an integrated approach to microbial environmental monitoring in cultural heritage: Experience at the Correggio exhibition in Parma. Aerobiologia.

[17] Rivas-Martinez S. (1996). Clasificacion Bioclimatica dela tierra. Folia Bot. Matr..

[18] Blasi C., Carranza M.L., Filesi L., Tilia A., Acosta A. (1999). Relation between climate and vegetation along a Mediterranean-Temperate boundary in central Italy. Glob. Ecol. Biogeogr..

[19] Herrero B., Fombella-Blanco M.A., Fernández-González D., Valencia-Barrera R.M. (1996). Aerobiological study of fungal spores from Palencia (Spain). Aerobiologia.

[20] Pelizzari F. (1996). Gravimetric survey of airborne fungal spores in Milan. Aerobiologia.

[21] Munuera M., Carrion J.S., Navarro C. (2001). Airborne Alternaria spores in SE Spain (1993–1998)—Occurrence patterns, relationship with weather variables and prediction models. Grana.

[22] Pyrri I., Kapsanaki-Gotsi E. (2007). A comparative study on the airborne fungi in Athens, Greece, by viable and non-viable sampling methods. Aerobiologia.

[23] Ruga L., Bonofiglio T., Sgromo C., Orlandi F., Romano B., Fornaciari M. (2007). Presenza di biodeteriogeni nel percorso museale “Il Perugino” e possibili relazioni con l’indoor e l’outdoor. Boll. ICR.

[24] Ascione F., Minichiello F. (2010). Microclimatic control in the museum environment: Air diffusion performance. Int. J. Refrig..

[25] Sedlbauer K. (2002). Prediction of Mould Growth by Hygrothermal Calculation. J. Therm. Envel. Build. Sci..

[26] Zorpas A.A., Skouroupatis A. (2016). Indoor air quality evaluation of two museums in a subtropical climate conditions. Sustain. Cities Soc..

[27] Caneva G., Nugari M.P., Salvadori O. (1991). Biology in the Conservation of Works of Art.

[28] Gallo F. (1992). Il Biodeterioramento di Libri e Documenti.

[29] Nyuksha Y.P., Garg K.L., Garg N., Mukerji K.G. (1994). The biodeterioration of paper and books. Recent Advances in Biodeterioration and Biodegradation.

[30] Zyska B. (1997). Fungi isolated from library materials: A review of the literature. Int. Biodeterior. Biodegrad..

[31] Valentin N., Saiz-Himenez C. (2003). Microbial contamination in museum collection: Organic material. Molecolar Biology and Cultural Heritage, Proceedings of the International Congress on Molecolar Biology and Cultural Heritage, Sevilla, Spain, 4–7 March 2003.

